# Assessment and management of frailty during pulmonary rehabilitation: An international survey of Australian and New Zealand clinicians

**DOI:** 10.1177/14799731251400252

**Published:** 2025-11-14

**Authors:** AL Alzubaidi, S Soh, M Wuyts, P Munro, KD Hill, CR Osadnik

**Affiliations:** 1 Department of Physiotherapy, 2541 Monash University, Melbourne, VIC, Australia; 2 Department of Physiotherapy, King Abdulaziz University, Jeddah, Saudi Arabia; 3 Department of Rehabilitation Sciences, KU Leuven, Leuven, Belgium; 4 Department of Rehabilitation Sciences, Ghent University, Ghent, Belgium; 5 Rehabilitation Ageing and Independent Living (RAIL) Research Centre, 2541 Monash University, Melbourne, VIC, Australia

**Keywords:** frailty, pulmonary rehabilitation, chronic lung disease

## Abstract

**Background:**

Frailty is common among patients referred to pulmonary rehabilitation (PR) programs. While it is considered an independent predictor of program non-completion, people with frailty respond positively to PR. Clinicians’ perspectives on assessing and managing frailty in PR has not been established. This study aimed to identify clinicians’ current practices, perceptions, and opinions on assessing and managing frailty in people who attend PR in Australia and New Zealand.

**Methods:**

An international online survey targeting healthcare professionals in Australia and New Zealand involved in PR programs using a combination of multiple-response closed-ended and open-ended questions. This survey study was reported in accordance with the Consensus-Based Checklist for Reporting of Survey Studies (CROSS).

**Results:**

Of 103 responses, 89 healthcare professionals completed the survey (92.6% completion rate). Nineteen percent routinely assessed frailty, mostly using Short Physical Performance Battery (SPPB). Respondents reported the main considerations for choice of frailty assessment tools were ease of use and cost. The most common frailty indicators identified by respondents included falls history, low body weight, slow or aided gait, and muscle weakness. Seventy-nine percent believed PR to be appropriate to manage frailty in this population, while 94% desired additional resources in future guidelines. Suggestions to improve PR to better manage frailty included reshaping rehabilitation program content, and providing specific patient education. Future desired research priorities included improvements to frailty assessment tools, frailty-specific guidelines and workforce training.

**Conclusion:**

This study explores PR practices among clinicians in Australia and New Zealand, showing variability in frailty assessment and management. It provides a foundation for evaluating key aspects of PR models that can be tailored to these clients’ needs and limitations.

## Introduction

Pulmonary rehabilitation (PR) is an essential non-pharmacological treatment for a range of chronic lung diseases. It is a multi-component and multidisciplinary therapeutic intervention that reduces symptoms, enhances function, and improves the quality of life of people with chronic lung diseases.^1,2^ As such, PR is recommended in international clinical practice guidelines,^3^ including in Australia and New Zealand.^1^

Frailty is a syndrome characterised by diminished reserves and heightened susceptibility to stressors.^4,5^ One of the most common models for conceptualising frailty describes it through five main clinical characteristics: unintentional weight loss, fatigue, muscle weakness, decreased gait speed, and reduced physical activity.^5^ The presence of frailty has been shown to be associated with adverse health outcomes for older adults, such as falls, acute care hospitalisations, disability and death.^4,5^ The global prevalence of frailty among patients with chronic lung disease ranges from 20% to 58%.^6–9^ Data from Australia and New Zealand are limited. However, one previous study attempted to measure how frailty affects long-term survival after intensive care unit (ICU) admission for Chronic Obstructive Pulmonary Disease (COPD) exacerbation, finding a frailty prevalence of 54%.^10^ Frailty has gained increased recognition as a “treatable trait”^11^ of importance in the management of people with chronic lung diseases.^12,13^ This has led to understandable inquiry regarding the suitability of PR programs to address the limitations and needs of people with chronic lung disease and coexisting frailty.

An emerging body of research examining the prevalence of frailty specifically within the PR context suggests it is potentially detectable in 25–58% of patients.^12,13^ Although limited data exists from robust clinical trials regarding the impact of PR on people with chronic lung disease and coexisting frailty, various cohort studies have shown frailty instruments demonstrate responsiveness to PR^14–19^ suggesting probable benefits. Despite this, gaps in knowledge remain regarding the role of PR in managing frailty as well as an understanding of current practices that clinicians use for assessing and managing frailty within PR. This is important to understand as the healthcare professionals responsible for delivering PR (e.g. physiotherapists, exercise physiologists, nurses, occupational therapists, dietitians and medical clinicians),^1–3^ may differ in their understanding and/or perspectives on how to ideally manage coexisting frailty in this context. Understanding the needs and perspectives of these healthcare professionals and the potential barriers and facilitators impacting upon service delivery are critical first steps towards improve clinical care for this important patient group.

## Objective

This study examined clinicians’ practices and views on assessing and managing frailty during PR in Australia and New Zealand.

## Methods

### Study design and survey development

This study was a cross-sectional electronic survey conducted between June to September 2021. Ethics approval was obtained from the Monash University Human Research Ethics Committee (reference 27053). Consent for anonymous participation was obtained within the survey following review of the explanatory statement. The survey involved 32 questions covering four areas: (1) demographic details of participants and PR programs; (2) the significance of assessing and managing frailty in these programs; (3) clinicians’ confidence in assessing and managing frailty during PR; and (4) specialised training in frailty management undertaken. Questions about the future of frailty assessment and management in PR were also included. To ensure the study’s brevity, most questions were designed using 5-point Likert scales (e.g., ‘much less effective,’ ‘somewhat less effective,’ ‘neutral/about the same,’ ‘somewhat more effective,’ and ‘much more effective’), multiple-response questions, or yes/no/unsure options. Open-ended questions were also included to allow for more in-depth responses. The survey was pilot-tested with five clinically-based allied health professionals to evaluate face validity. This resulted in minor wording adjustments for clarity and suggestions to reduce total survey length, which were accommodated. A blank copy of the final instrument is included in the Supplemental S-1. The survey was constructed in a university-hosted secure Qualtrics account, accessible only to the project team. Default settings were applied without imposing additional restrictions on IP addresses (e.g. to prevent survey re-entry or unintended multiple completions by individuals). Reporting of findings followed the Consensus-Based Checklist for Reporting of Survey Studies (CROSS) checklist.^20^ Supplemental S-2: Completed CROSS Checklist.

### Settings and participants

Healthcare professionals working in PR who were involved in the coordination or delivery of PR in the last 24 months were eligible to participate. The research team contacted participants via email, social media outlets and advertisements in peak respiratory bodies across Australia and New Zealand (retained on message boards throughout the study period). Participants were encouraged to circulate the survey link among interested colleagues (i.e., snowball sampling). The survey was expected to take fifteen to 20 min. No formal power calculation was conducted to determine a target sample size due to the exploratory and broad nature of the survey and the lack of suitable data upon which to formulate such decisions. Due to the snowballing nature of recruitment, no follow-up action was able to be conducted at the individual participant level, and response rates were not able to be calculated.

### Data analysis

Survey data were analysed using Stata 14 SE. Categorical data were expressed in terms of frequencies and percentages, illustrated in appropriate graphs or tables. Responses from five-point Likert scales were dichotomised into two categories: (‘much more effective/somewhat more effective’ and ‘about the same/somewhat less effective/much less effective’) permitting comparisons via Chi-square test. No attempts were made to manage or impute missing data apart from conducting a complete analysis of available data per item (e.g. relative to the denominator per question). No sensitivity analyses were conducted or attempts made to adjust variable data or account for any confounders.

Open-ended responses were synthesised using a simplified process of content analysis.^21^ Briefly, this involved familiarisation of the dataset through repeated readings, coding of recurring ideas and concepts, collation of data under preliminary themes, and refinement to determine final theme descriptors. Themes were initially identified and developed by author AA, then independently reviewed and discussed with authors CO and KH to ensure consensus and reliability. Cases lacking responses to these questions were omitted from the analysis.

## Results

### Demographic data

Surveys were distributed to 56 individual contacts of the research team and distributed to the national network of registered PR programs in Australia and New Zealand. Of 103 participants who commenced the survey, 89 healthcare professionals completed the survey (93% completion rate). Seventy-two percent of the responses (64/89) were from Australia, while 28% (25/89) were from New Zealand. Respondents represented various disciplines in PR, with the majority being physiotherapists 61% (54/89) and nurses, 29% (26/89). See Table 1 for details of participant demographics.

**Table 1.** Demographic characteristics of participants from PR settings.

Demographic factors	*N*		*n* (%)
Country	89	Australia	64 (72%)
		New Zealand	25 (28%)
Australian state	64	Australian Capital Territory	2 (3%)
		New South Wales	13 (20%)
		Queensland	12 (19%)
		South Australia	9 (14%)
		Tasmania	1 (2%)
		Victoria	18 (28%)
		Western Australia	9 (14%)
New Zealand region	25	Auckland	8 (32%)
		Waikato	2 (8%)
		Bay of Plenty	1 (4%)
		Hawke’s Bay	3 (12%)
		Manawatu-Wanganui	1 (4%)
		Wellington	4 (16%)
		Cantebury	3 (12%)
		Otago	1 (4%)
		Nelson-Tasman	1 (4%)
		Marlborough	1 (4%)
Gender	89	Man	9 (10%)
		Woman	80 (90%)
Profession	89	Physiotherapist	54 (61%)
		Exercise physiologist	3 (3%)
		Occupational therapist	3 (3%)
		Nurse	26 (29%)
		Dietitian/Nutritionist	1 (1%)
		Medical practitioner	1 (1%)
		Other	1 (1%)
Highest qualification	89	Workforce entry degree	61 (69%)
		Clinical Masters	9 (10%)
		MPhil	6 (7%)
		PhD	6 (7%)
		Other	7 (8%)
Role	89	Initial assessment	80 (90%)
		Re-assessment	70 (79%)
		Coordinator	52 (58%)
		Patient educator	75 (84%)
		Assessment as needs	61 (69%)
		Facilitator therapist	67 (75%)
		Median (IQR)	
Years of practice	89	17 (9, 17)	
Years in PR	89	8 (4, 16)	

Abbreviations: PR: pulmonary rehabilitation; MPhil: master of philosophy; PhD: doctor of philosophy; N: total sample size; n: subgroup sample size; IQR: interquartile range.

Table 2 shows the characteristics of the PR program which respondents coordinated or delivered. Eighty-eight per cent (78/89) were public sector programs; the remaining were from the private sector. Programs were primarily outpatient 57% (51/89), or community-based 53% (47/89), with 26% (23/89) home-based, and 25% (22/89) telehealth (some respondents mentioned multiple settings).

**Table 2.** Demographic data (PR programs).

Demographic factor	*N*	Settings	*n* (%)
Sector	89	Public	78 (88%)
		Private	11 (12%)
Settings^a^	89	Inpatient program	5 (6%)
		Outpatient program	51 (57%)
		Community-based program	47 (53%)
		Home-based program	23 (26%)
		Tele-rehab program	22 (25%)
		Median (IQR)	
Total PR length (weeks)	89	8 (8, 8)	
Number of PR (sessions per week)	89	2 (2, 2)	
Session duration (mins)	89	90 (60, 120)	

Abbreviations: PR: pulmonary rehabilitation; N: total sample size; n: subgroup sample size; IQR: interquartile range.

^a^Percentages may exceed 100% because respondents were able to select multiple. Outpatient programs involve scheduled, clinical treatment at facilities like—hospitals or clinics, focusing on medical or therapeutic care. Community-based programs offer flexible, non-clinical support in local settings—like community centres, schools, churches, or even patients’ homes, emphasising social integration and peer-led assistance.

### Current practice

**Figure 1.**
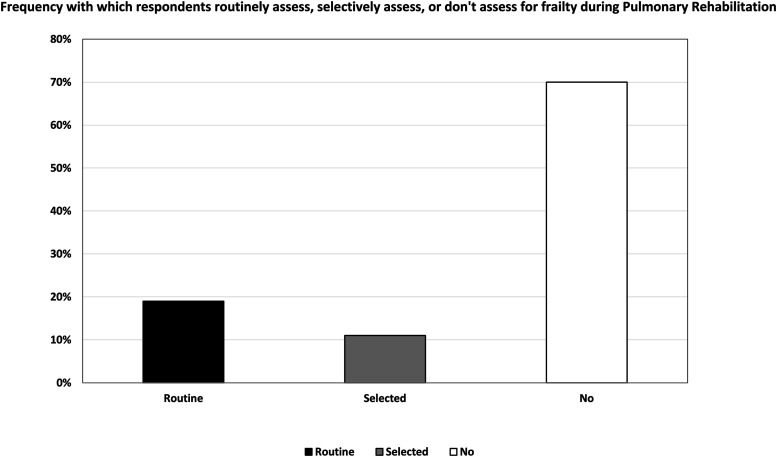
Frequency with which respondents routinely assess, selectively assess, or don’t assess for frailty during Pulmonary Rehabilitation. Figure 1 Caption: *Out of 89 responses to the question “Do you routinely assess for the presence of frailty in people who present to pulmonary rehabilitation with chronic lung diseases?”, 17 (19.1%) indicated routine screening for all people, 10 (11.2%) screen only select individuals, and 62 (69.7%) do not assess frailty at all.*

**Figure 2.**
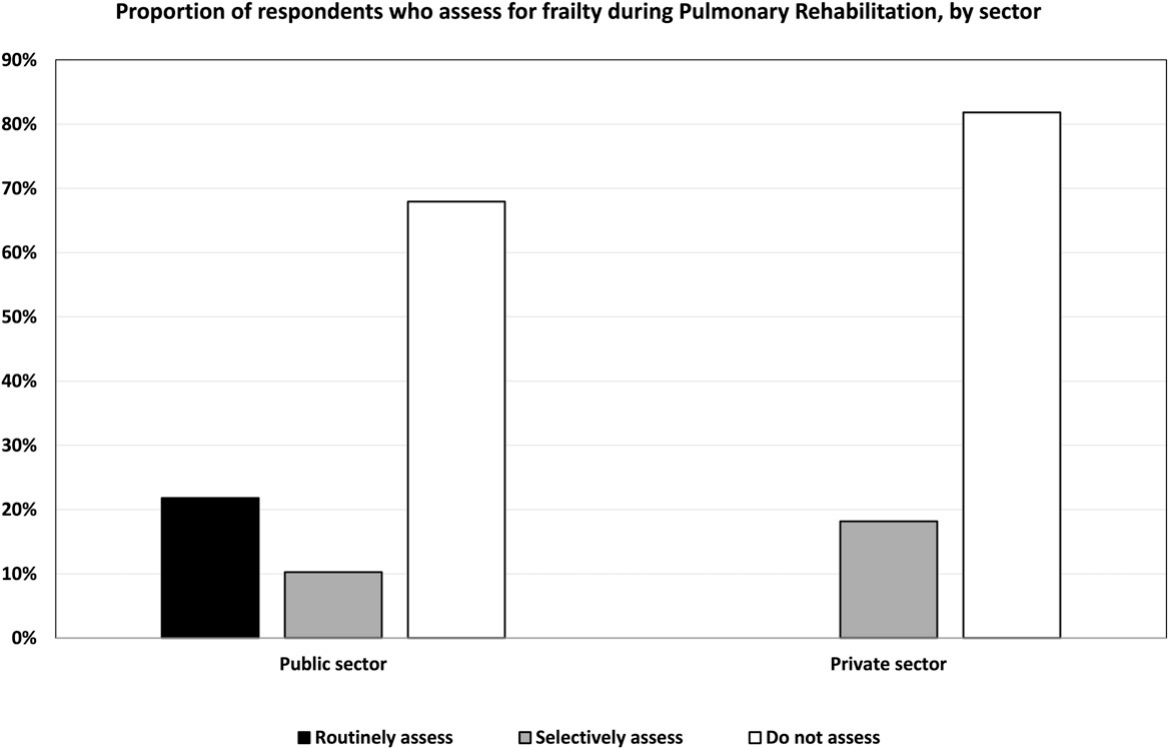
Proportion of respondents who assess for frailty during Pulmonary Rehabilitation, by sector. Figure 2 Caption: *A difference in the use of routine frailty assessment appeared between public and private sector sites, occurring only in public health services; however, this was not statistically significant (p-value = 0.202)*.

**Figure 3.**
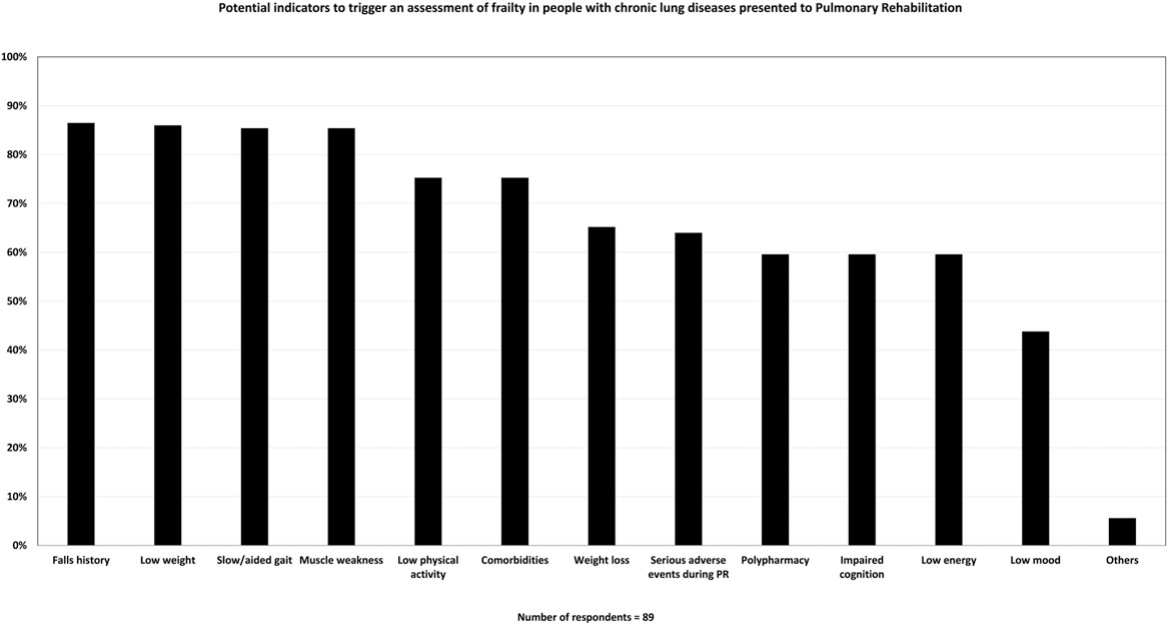
Potential indicators to trigger an assessment of frailty in people with chronic lung diseases presented to Pulmonary Rehabilitation.

Nineteen percent (17/89) of respondents reported they routinely assessed frailty during PR (Figure 1). Interestingly, there appeared a difference in the use of routine frailty assessment between public and private sector sites with it only occurring in public health services, however this were not statistically significant (*p* = 0.202; Figure 2). The use of specific instruments to assess frailty was varied and low in general. The most commonly used instruments were: Short Physical Performance Battery (SPPB): 6% (5/89); Clinical Frailty Scale (CFS): 4% (4/89); FRAIL: 3% (3/89); Fried Frailty Phenotype (FFP): 2% (2/89); and Frailty Index (FI): 2% (2/89). The reasons respondents selected a specific assessment tool over others also varied, with the most reported factor being the ease of using the instrument (ease of administering the tool, ease for clinicians to understand/administer, and/or ease for patients to understand/perform), as indicated by 38% (34/89) of respondents. As 14.6% (13/89) of respondents indicated, low cost also influenced their tool selection. Other reasons included centre protocols, no training required, equipment availability, widespread usage, no-cost licensing, and evidence-based support, which together accounted for 40% of responses—(34/89) selected one or more of these options. However, only 4.5% (4/89) of respondents attributed sensitivity to detecting change during PR as the reason for their selected instrument (see Supplemental S-3). The most common indicators for assessing frailty reported by participants were evidence of a fall history 86.5% (77/89), low weight 86.5% (77/89), slow or aided gait 85.4% (76/89), and muscle weakness 85.4% (76/89) (Figure 3).

### Perceptions regarding PR to manage frailty

**Figure 4.**
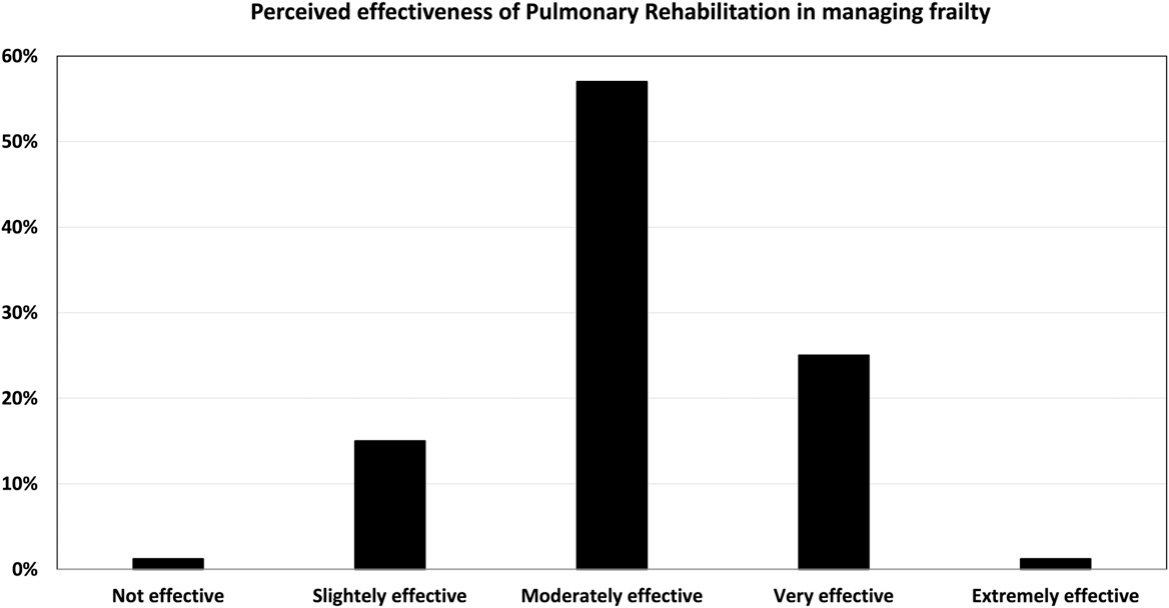
Perceived effectiveness of Pulmonary Rehabilitation in managing frailty.

Table 3 summarises various clinician perspectives. The majority of respondents, 94% (68/72), desired to see specific advice and/or training resources for managing frailty integrated within future PR guidelines and/or training resources, although 6% (4/72) indicated they were unsure (Table 3). No differences were apparent between clinicians who had less than or greater than 5 years experience in PR (*p* = 0.715) (Table 3). Twenty six percent (22/84) of respondents rated PR as very or extremely effective for the management of frailty (Figure 4); however, there was no clear evidence that clinicians were confident in their ability to assess or manage frailty. This pattern did not differ between clinicians with less than or more than 5 years experience in PR (*p* = 0.279 for assessment; *p* = 0.692 for management).

**Table 3.** Respondents’ perceptions regarding frailty assessment and management in PR settings.

Perceptions and opinions on	***N***		*n* (%)
Confidence to assess frailty	72	Confident	38 (53%)
		Not confident	34 (47%)
Confidence to manage frailty	72	Confident	40 (56%)
		Not confident	32 (44%)
Importance of frailty-specific strategies	72	Important	46 (64%)
		Not important	26 (36%)
Desire for new resources *(specific advice and/or training resources for managing frailty integrated within future PR guidelines and/or training resources)*	72	Yes	68 (94%)
		Unsure	4 (6%)
Receive sufficient training re: frailty	71	Yes	7 (10%)
		No	52 (73%)
		Unsure	12 (17%)
Training appropriate to improve healthcare professionals’ knowledge	71	Accredited post-grad degree	3 (4%)
		Embed in future graduate training	35 (49%)
		Professional development	64 (90%)
		Self-directed learning	45 (63%)
		Seminar event	39 (55%)
Suitability of PR for frailty	70	Appropriate	55 (79%)
		Not appropriate	15 (21%)
Difficulty to adapt PR to frailty	70	Easy	22 (31%)
		Not easy	48 (69%)

Abbreviations: PR: pulmonary rehabilitation; N: total sample size; n: subgroup sample size; IQR: interquartile range.

Participants identified several barriers to assessment and management (Table 4). Lack of awareness among healthcare professionals was a major issue, with 56% (50/89) citing it as a barrier to assessment and 52% (46/89) to management. Lack of training was reported by 56% (50/89) for assessment and 53% (47/89) for management. The absence of current recommendations in PR guidelines was noted by 60% (53/89) for assessment and 56% (50/89) for management. Time constraints impacted 58% (52/89) in assessment and 29% (26/89) in treatment, while lack of confidence in assessing frailty was reported by 55% (49/89) for assessment, and 28% (25/89) saw it as a barrier for management.

**Table 4.** Barriers to assessment and treatment of frailty during PR.

**N = 89**			
Barriers to assessment	*n* (%)^a^	Barriers to treatment	*n* (%)
Awareness	50 (56%)	Awareness	46 (52%)
Lacking confidence	49 (55%)	Lacking confidence	25 (28%)
Insufficient funds	28 (31%)	Insufficient funds	34 (38%)
Time	52 (58%)	Time	26 (29%)
Training needs	50 (56%)	Training needs	47 (53%)
Lack of guidelines	53 (60%)	Lack of guidelines	50 (56%)
Too many tools	52 (58%)	Too many tools	17 (19%)

Abbreviations: PR: pulmonary rehabilitation.

^a^Percentages may exceed 100% because respondents were able to select multiple.

### Aspects of PR requiring adapting

**Figure 5.**
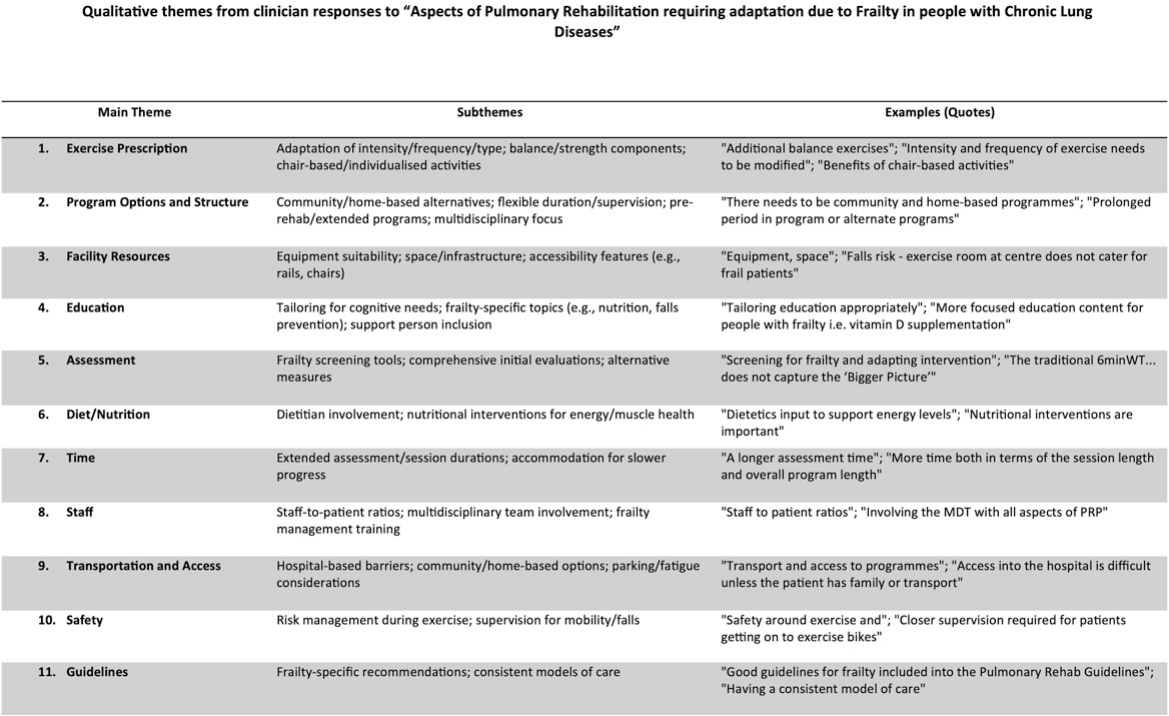
Qualitative themes from clinician responses to “Aspects of Pulmonary Rehabilitation requiring adaptation due to Frailty in people with Chronic Lung Diseases”.

Clinicians identified multiple aspects of PR programs they felt required adaptation to address frailty in people with chronic lung disease. These are described in detail in the Supplemental S-4 and Figure 5. Example topics included: exercise prescription (e.g., tailored intensity and balance components), education (e.g., cognitive adaptations and frailty-specific content), safety (e.g., fall risk management), staffing (e.g., improved ratios and multidisciplinary input) and program structure (e.g., flexible duration, support for community transport).

### Priorities areas for future work related to frailty in PR

**Figure 6.**
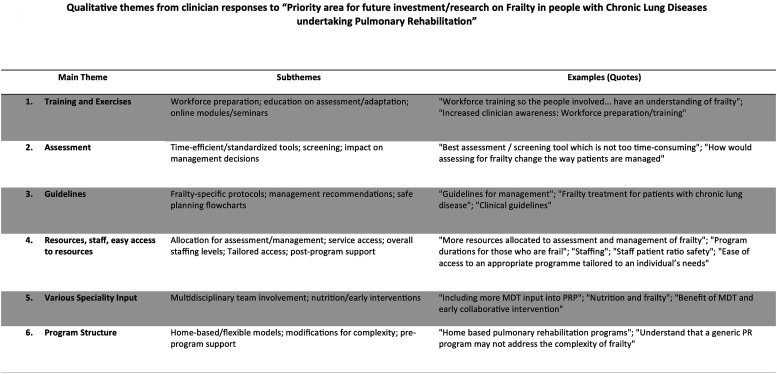
Qualitative themes from clinician responses to “Priority area for future investment/research on Frailty in people with Chronic Lung Diseases undertaking Pulmonary Rehabilitation”.

Several multifaceted aspects of PR were perceived to require future research to enhance the management of frailty. These included improvements to assessment methods (e.g., standardisation of tools), guideline development (e.g., protocols for exercise prescription and progression), program structure (e.g., flexible models, multidisciplinary team input, staff ratios), and future education (e.g., of patients and the health workforce). Further information regarding these perspectives is available in Figure 6 and Supplemental S-5.

## Discussion

Pulmonary rehabilitation (PR) is an essential component in the management of a range of chronic lung diseases; however, its precise role in addressing the complex needs of individuals affected by coexisting frailty has yet to be fully defined. Frailty is an important “treatable trait”^11^ and PR appears to address some of the potentially contributing factors underpinning its development. However, the limited evidence in this field^17^ means gaps in knowledge and clinical guidance exist, which can lead to clinical uncertainty and service variability. This underpins the importance of the present study to seek perspectives from clinicians at the forefront of this important area of practice. While contextualised to Australia and New Zealand respondents, the findings are likely transferable to many other settings with similar health systems and PR program structures.

It was interesting to note that most respondents felt they lacked adequate training in the assessment and management of frailty in people with chronic lung diseases, while 70% (62/89) of clinicians reported not assessing frailty during PR, despite its high reported prevalence.^6,17,22–26^ Further, frailty assessment were only routinely conducted in public and not private health settings. While the reasons for this are not clear, it is plausible frailty awareness could be greater in settings attached to hospital-based healthcare settings (e.g. outpatient departments) due to educational opportunities that might traverse such boundaries. Of the instruments used to evaluate frailty, the SPPB was the most frequently reported tool. The SPPB demonstrates good responsiveness to PR interventions in multiple studies,^14–19^ and its preference for use might reflect this rationale. Alternatively, it may also be perceived a simple tool to implement or may be a familiar tool to clinicians and/or one that pertains relevance to aspects of treatment decision-making. It is multi-component in nature, but focuses on physical performance and thus the concept of ‘physical frailty’. If considering frailty through a broader lens, one might argue use of the SPPB neglects the necessary dimensionality to gain accurate insights into patients’ individual contributing factors to their condition (e.g. cognition, environmental and/or psychosocial domains). This poses a challenging clinical dilemma as broader instruments possessing a more comprehensive focus to one’s frailty may impose time cost burdens for implementation and lack sensitivity to change following an intervention such as PR. Examples of such tools, such as the Edmonton Frailty Scale,^27^ Groningen Frailty Indicator (GFI)^28^ or Tilburg Frailty Indicator (TFI),^29^ were rarely used by survey participants. No specific instrument has yet been advocated for use as a ‘gold standard’ in PR. There may, however, be benefit in exploring the roles of different instruments to determine the potential to better tailor personalised treatments during PR based on the underlying frailty components that might exist within individuals.

Almost 75% (66/89) of survey participants perceived PR as not being extremely or very effective for managing frailty in this population (Figure 4). This appears at odds with previous studies that suggest individuals with very poor exercise tolerance actually have the greatest potential and magnitude for improvement from PR.^17,30^ Several factors could explain this observation. One may relate to the increasing multi-systemic clinical complexity inherent in frailty, which can contribute, almost by definition, to various physical, social, and psychological challenges to engage with and derive benefit from.^31^ Another factor can be the downstream impacts of frailty on access to services, transport, and facilities, which can hinder progress. This may also relate to findings indicating frailty as an independent predictor of program non-completion.^17^ An alternative explanation for this perception could be a lack of experience or knowledge by clinicians about how to manage frailty in a PR setting, indicated by respondents’ reported difficulty in adapting PR to frailty 69% (48/70), insufficient training 73% (52/71), a strong desire for new resources 94% (68/72), and perceived barriers such as low awareness, lack of confidence, and absence of guidelines (Table 3). This suggests a possible need to develop and disseminate resources and training for clinicians to improve their knowledge, skills and confidence in managing frailty optimally in people undergoing PR. These could take the form of informal ‘professional development’ resources through professional societies or more formalised training courses/credentials through specialist centres or education providers. Ultimately, it will be important that future practice guidelines incorporate necessary adaptations to account for the unique issues imposed by frailty and that quality improvement measures or standards are considered to ensure effective implementation.

It is interesting to speculate how these insights might impact future clinical practice. Important perspectives that are presently lacking include the patient and caregiver perspectives on frailty during PR. This is an important gap worthy of further research. Another interesting issue is the ‘fit’ of PR for the management of frailty in people with chronic lung disease. On the one hand, frailty is now well recognised as a treatable trait of importance, and PR is arguably one of the leading interventions to help address it due to its multidisciplinary focus and ability to target the physical components of frailty. However, this does not mean PR effectively manages all aspects of frailty nor that all individuals who participate in PR gain the optimal benefits from PR. Survey respondents in the present study suggested adaptations may be indicated to better account for the issues unique to frailty, and it is plausible that conventional approaches to exercise training might not ideally suit the limitations specific to this patient sub-group. Further, the high dropout rate previously highlighted in people with COPD and frailty^17^ suggests the PR model itself might require careful consideration to ensure optimal engagement and completion. This has been highlighted previously.^12,13,32^

## Limitations

The present study is not without some limitations. Our use of snowballing sampling of clinicians meant determining an accurate response rate relative to the total potential sample pool is not possible. Our distribution to PR coordinators and clinicians in the field may also have skewed responses mostly to physiotherapists (who most commonly coordinate these services in Australia and New Zealand) despite the goal to sample multidisciplinary staff more broadly. While we were unable to determine an accurate response rate for this survey, we note the 89 responses compare favourably to prior national efforts^33,34^ which similarly yielded informative perspectives on the health workforce landscape. Despite these limitations, the study offers valuable insights into clinicians’ perspectives on frailty assessment and management during PR that have not previously been explored. Additionally, while consumer input from patients with frailty would have been desired, this was deemed beyond the scope of the present study remit.

## Conclusion

Clinical practice for the assessment and management of frailty during PR in Australia and New Zealand is varied and requires greater focus and support in order to embed systematic improvements aimed at improving clinical outcomes for this important patient group. Several key areas for future improvement were highlighted that could serve as a useful foundation for future research.

## Supplemental Material

Suppplemental Material - Assessment and management of frailty during pulmonary rehabilitation: An international survey of Australian and New Zealand clinicians Suppplemental Material for Assessment and management of frailty during pulmonary rehabilitation: An international survey of Australian and New Zealand clinicians by AL Alzubaidi, S Soh, M Wuyts, P Munro, KD Hill, CR Osadnik in Chronic Respiratory Disease
