# Supplementary material for: Assessment and management of frailty during pulmonary rehabilitation: An international survey of Australian and New Zealand clinicians
Source: Chron Respir Dis. 2025 Nov 14;22:14799731251400252. doi: 10.1177/14799731251400252 (PMC12618826; doi:10.1177/14799731251400252)
Supplement: Suppplemental Material - Assessment and management of frailty during pulmonary rehabilitation: An international survey of Australian and New Zealand clinicians [file sj-pdf-1-crd-10.1177_14799731251400252.pdf]

Assessment and management of frailty during pulmonary rehabilitation: an international survey of Australian and New Zealand clinicians

Supplementary Materials

Authors:

Alzubaidi AL, Soh S, Wuyts M, Munro P, Hill KD, Osadnik CR.

Contributor/acknowledgement:

Anita Stieglbauer, Melbourne, Australia

Contents:

- **Supplementary S-1:** Blank Copy of the Survey
- **Supplementary S-2:** Completed CROSS Checklist
- **Supplementary S-3:** Table 5, Reasons to assess for frailty
- **Supplementary S-4:** Aspects of Pulmonary Rehabilitation require adaptation for Frailty
- **Supplementary S-5:** Priorities for future work
- **Supplementary S-6:** Free comments or insights

Supplementary S-1: Blank Copy of the Survey

Frailty in pulmonary rehabilitation programs: an Australian and New Zealand survey

Thank you for your time and interest in this survey examining current practice and perspectives of healthcare professionals working in Australian and New Zealand pulmonary rehabilitation programs regarding the assessment and management of people who have chronic lung diseases and frailty.

For the purpose of this study, **Chronic lung diseases refer** to diseases of the airways and other lung structures that prevent the lungs from functioning properly, such as chronic obstructive pulmonary disease (COPD), bronchiectasis, interstitial lung diseases and pulmonary hypertension. **Frailty refers to** an age-related accumulation of deficits leading to increased vulnerability to stressors that predisposes affected individuals to adverse health consequences.

This project has been granted ethics approval from the Monash University Human Research Ethics Committee (reference #27053).

Before deciding whether to voluntarily participate in this study, we encourage you to view the full project Explanatory Statement, which is available by clicking HERE.

The survey is anticipated to take approximately 15-20 minutes to complete and all responses are anonymous. You will have the option to participate in a follow-up project at the end of the survey. If this is something you would like to assist with, you will be asked to enter your contact details in a separate Qualtrics link (not linked to results from this survey). The next section will determine your eligibility to participate in the survey.

Q1. Eligibility: **Are you a qualified healthcare professional who has provided a pulmonary rehabilitation service to people with chronic lung diseases during the past 24 months?**

This must include either the direct assessment of patients and/or the delivery of exercise, dietary advice counselling, medication prescription etc. Please note, this *excludes* healthcare professionals who refer patients to pulmonary rehabilitation but are not involved in its delivery.

1. Yes
2. No

Section 1 - About you

Q2. With which gender do you identify?

1. Man
2. Woman
3. Non-binary / gender diverse
4. My gender is not listed
5. Prefer not to say

Q3. What is your profession?

1. Physiotherapist
2. Exercise Physiologist
3. Occupational Therapist
4. Nurse
5. Dietitian/Nutritionist
6. Social Worker
7. Psychologist
8. Pharmacist
9. Medical Practitioner
10. Other - Please specify ____

Q4. What is your role in the pulmonary rehabilitation program?

1. Conduct initial assessments

2. Conduct re-assessments

3. Program coordinator

4. Patient educator

5. Provide individual patient assessments as required (e.g. on a referral basis)

6. Session facilitator/therapist

Q5. For how many years have you worked in your profession?

1 <1 year

2. =1 year

3. =2 years

4. =3years

5. =4years

6. =5years

7. =6years

8. =7years

9. =8years

10. =9years

11. =10years

12. =11years

13. =12years

14. =13years

15. =14years

16. =15years

17. =>15years

Q6. For how many years have you worked in pulmonary rehabilitation?

1. <1 year

2. =1 year

3. =2 years

4. =3years

5. =4years

6. =5years

7. =6years

8. =7years

9. =8years

10. =9years

11. =10years

12. =11years

13. =12years

14. =13years

15. =14years

16. =15years

17. =>15years

Q7. What is your highest level of qualification to practice in your field?

1. Primary workforce entry professional degree (e.g. Bachelors, Graduate entry Masters, Masters extended etc.)

2. Post-graduate clinical masters specialization degree (e.g. Masters via coursework such as a Master of Sports)

3. Post-graduate research masters degree (e.g. Master of Philosophy)

4. Post-graduate professional/clinical doctorate specialization degree (e.g. coursework oriented doctorate (Not PhD)

5. Post-graduate research doctorate (e.g. Doctor of Philosophy)

6. Other (please specify) ____

Q8. In which country are you based?

1. Australia
2. New Zealand

Q9. In what Australian state or territory do you work?

1. Australian Capital Territory
2. New South Wales
3. Northern Territory
4. Queensland
5. South Australia
6. Tasmania
7. Victoria
8. Western Australia

Q10. In what New Zealand region do you work?

1. Northland
2. Auckland
3. Waikato
4. Bay of Plenty
5. Gisborne
6. Hawke's Bay
7. Taranki
8. Manawatu-Wanganui
9. Wellington
10. West Coast
11. Canterbury
12. Otago

13. Southland

14. Nelson-Tasman

15. Marlborough

Section 2 – About your pulmonary rehabilitation program

This section includes questions related to the pulmonary rehabilitation program that you are involved with. It includes questions about the sessions and the different therapy components that are offered within your program. If you deliver pulmonary rehabilitation across multiple programs (e.g. different geographic sites) or settings (e.g. home-based, outpatient), please either answer with respect to ANY of them or the one you feel is your principal program.

Q11. In which health sector is your pulmonary rehabilitation based?

1. Private sector
2. Public sector

Q12. In which setting(s) does your pulmonary rehabilitation program operate?
Select all that apply.

1. Hospital inpatient
2. Hospital outpatient
3. Community
4. Home
5. Telerehabilitation
6. Other (please specify)

Q13. For how many weeks does your program run?

1. =1week

2. =2weeks

3. =3weeks

4. =4weeks

5. =5weeks

6. =6weeks

7. =7weeks

8. =8weeks

9. =9weeks

10. =10weeks

11. =11weeks

12. =12weeks

13. =13weeks

14. =14 weeks

15. =15 weeks

16. =16 weeks

17. =17 weeks

18. =18 weeks

19. =19 weeks

20. =20 weeks

21. =>20 weeks

Q14. How many sessions per week are scheduled for your pulmonary rehabilitation program?

In answering this, please consider 'sessions' to equate to pre-planned patient interactions with healthcare professional and/or their peers. This will commonly refer to physical attendance at centre-based programs or remotely supervised off-site sessions (e.g. telerehabilitation), but should not refer to unsupervised home exercise program sessions.

1. =1session

2. =2sessions

3. =3sessions

4. =4sessions
5. =5sessions
6. =6sessions
7. =7sessions
8. =8sessions
9. =9sessions
10. =10sessions
11. =11sessions
12. =12sessions
13. =13sessions
14. =14sessions
15. =>14sessions

Q15. Approximately how long does each session of your pulmonary rehabilitation program last (inclusive of exercise +/- any other components)?

1. =10minutes
2. =20minutes
3. =30minutes
4. =40minutes
5. =50minutes
6. =60minutes
7. =70minutes
8. =80minutes
9. =90minutes
10. =100minutes
11. =110minutes
12. =120minutes
13. =130minutes
14. =140minutes
15. =150minutes

16. >150minutes

<u>**Section 3 – Frailty Assessment and Management**</u>

This section focuses on issues related to frailty. Please answer the following questions irrespective of whether you assess or manage frailty in your clinical practice of your program.

Q16. Do you *routinely* assess for the presence of frailty in people who present to pulmonary rehabilitation with chronic lung diseases?

1. Yes (we screen all people)
2. No (we only screen in select individuals)
3. No (we do not assess frailty)

Q17. Below is a list of instruments reported in the literature for the assessment of frailty in clinical practice. Please indicate whether you use any of these in your clinical pulmonary rehabilitation practice.
Select all that apply.

1. Adelaide Frailty Index
2. Clinical Frailty Scale
3. Comprehensive Frailty Assessment Instrument
4. Edmonton Frailty Scale
5. Fried Frailty Phenotype
6. Frailty Index
7. FRAIL Scale
8. Groningen Frailty Indicator
9. Kihon checklist
10. Korean Frailty Index
11. Reported Edmonton Frail Scale

12. SHARE Frailty Instrument

13. Short Physical Performance Battery (SPPB)

14. Tilburg Frailty Indicator

15. Vulnerable Elders Scale-13 (VES-13)

16. Other (please specify) ____

Q18. Please tell us *why* you use the instrument(s) to assess frailty nominated in the prior question. Select all that apply.

1. Availability of normative reference data

2. Ease of access to equipment for conducting testing

3. Ease of administering the tool (e.g. quick, and simple)

4. Ease for clinicians to understand/administer

5. Ease for patients to understand/perform

6. Evidence based (e.g. data exists for chronic lung diseases and/or pulmonary rehabilitation)

7. Lack of need for license

8. Low/no cost of assessment tool

9. No training required of health professionals

10. Protocol at our centre

11. Sensitive to change as a result of pulmonary rehabilitation

12. Streamlined into existing technology (e.g. mobile apps, electronic medical record forms)

13. Translations available for our patients

14. Widely used/recommended

15. Other (please specify) ____

Q19. Which of the following factors do you feel are potentially useful indicators that might suggest a need for assessing frailty in people with chronic lung diseases who present to pulmonary rehabilitation? This can include factors you currently use for screening frailty or those that you feel could be useful to adopt. Select all that apply.

Patients who:

1. Are usually physically inactive
2. Have a history of polypharmacy
3. Have serious adverse events during pulmonary rehabilitation (e.g. falls, vaso-vagal episodes)
4. Present with a prior history of falls
5. Present with being underweight (e.g. low body mass index, malnourished, cachectic)
6. Present with muscle weakness
7. Present with multiple comorbidities
8. Report a history of unintentional weight loss
9. Report having reduced cognition
10. Report low energy levels
11. Report reduced mood
12. Walk slowly or requires assistance (e.g. gait aid)
13. Other ____

Q20. For people with chronic lung diseases who are typically referred to your program, how effective do you think pulmonary rehabilitation is at managing frailty?

1. Not effective at all
2. Slightly effective
3. Moderately effective
4. Very effective
5. Extremely effective

Q21. For people with chronic lung diseases who are typically referred to your program, please tell us how much you feel pulmonary rehabilitation impacts upon the following outcomes in those who have frailty *compared to those who do not have frailty*:

	Much less effective	Somewhat less effective	Neutral / about the same	Somewhat more effective	Much more effective
Balance	○	○	○	○	○
Education	○	○	○	○	○
Functional exercise tolerance	○	○	○	○	○
Future exacerbation / hospitalisation risk	○	○	○	○	○
Likelihood of patient attendance / program completion	○	○	○	○	○
Lower limb strength	○	○	○	○	○
Mental health (e.g. anxiety/depression)	○	○	○	○	○
Mortality	○	○	○	○	○
Physical activity levels	○	○	○	○	○
Quality of life	○	○	○	○	○
Self-management skills (e.g. smoking cessation/medication adherence)	○	○	○	○	○
Symptoms (e.g. shortness of breath, fatigue, etc.)	○	○	○	○	○
Upper limb strength	○	○	○	○	○

Q22. What do you feel are barriers to the effective *assessment* and *management* of frailty in people with chronic lung diseases who attend pulmonary rehabilitation? Please select all that apply for *assessment* and *management*, separately (please leave box unchecked if you <u>do not</u> feel it is a barrier).

	Barrier to assessment	Barrier to management
Lack of awareness amongst health care professionals	○	○
Lack of confidence to assess frailty	○	○
Lack of funding	○	○
Lack of time allocated for assessments	○	○
Lack of training amongst health care professionals	○	○
No current recommendations in pulmonary rehabilitation guidelines	○	○
Too many assessment tools	○	○
Other	○	○

Section 4 – Your views on frailty, chronic lung diseases and pulmonary rehabilitation

This section includes questions about your current opinions regarding frailty in people with chronic lung diseases who have frailty.

Q23. How confident do you feel to effectively *assess* and *manage* frailty in people with chronic lung diseases who attend pulmonary rehabilitation?

	Extremely unconfident	Somewhat unconfident	Neither unconfident or confident	Somewhat confident	Extremely confident
Assessment of frailty	o	o	o	o	o
Management of frailty	o	o	o	o	o

Q24. How important do you feel it is to have *assessment* and *management* strategies available that specifically cater for people with chronic lung diseases who have frailty during pulmonary rehabilitation?

1. Not important at all
2. Slightly important
3. Moderately important
4. Very important
5. Extremely important

Q25. Would you like to see specific advice regarding the management of frailty included within future pulmonary rehabilitation guidelines and/or training resources (e.g. the Pulmonary Rehabilitation Toolkit [https://pulmonaryrehab.com.au/])?

1. Yes
2. No
3. Unsure

Q26. Do you feel you received adequate health professional training regarding the *assessment* and *management* of frailty in people with chronic lung diseases who undertake pulmonary rehabilitation?

1. Yes
2. No
3. Unsure

Q27. What type of training do you feel would be most appropriate to improve healthcare professionals' knowledge regarding the *assessment* and *management* of frailty in people with chronic lung diseases who attend pulmonary rehabilitation?
Select all that apply.

1. Accredited degree (e.g. Diploma/Graduate Certificate/Postgraduate research degree)
2. Embed content into undergraduate training for future clinicians
3. Professional development modules(s)/workshops(s)/course(s)
4. Self-directed learning/research
5. Standalone lecture(s)/seminar(s)

Q28. How appropriate do you feel the pulmonary rehabilitation setting is for the comprehensive management of frailty in people with chronic lung diseases?

1. Very inappropriate
2. Somewhat inappropriate
3. Neutral
4. Somewhat appropriate
5. Very appropriate

Q29. How difficult do you find it to adapt your management of people with chronic lung diseases during pulmonary rehabilitation as a result of them having frailty?

1. Very difficult
2. Somewhat difficult
3. Neutral/No difference
4. Somewhat easy
5. Very easy

Q30. What aspects of pulmonary rehabilitation care do you feel require adapting as a result of people with chronic lung diseases having frailty?

____

____

____

____

____

Q31. If you could list one priority area for future investment/research regarding frailty in people with chronic lung diseases undertaking pulmonary rehabilitation, what would it be?

This can relate to any area of clinical practice, workforce preparation/training, patient-centred care, etc.

____

____

____

____

____

Q32. Please provide any free comments or insights regarding frailty in people with chronic lung disease and pulmonary rehabilitation in the text box below (optional).

____

____

____

____

____

<u>The end of the instrument</u>

Supplementary S-2: Completed CROSS Checklist

Checklist for Reporting Of Survey Studies (CROSS)

**Section/topic**	**Item**	**Item description**	**Reported on page #**
**Title and abstract**			
**Title and abstract**	1a	State the word “survey” along with a commonly used term in title or abstract to introduce the study’s design.	**Page 3, Line 22**
	1b	Provide an informative summary in the abstract, covering background, objectives, methods, findings/results, interpretation/discussion, and conclusions.	**Page 3, Lines 3—56**
**Introduction**			
**Background**	2	Provide a background about the rationale of study, what has been previously done, and why this survey is needed.	**Page 4, Lines 3—58**
**Purpose/aim**	3	Identify specific purposes, aims, goals, or objectives of the study.	**Page 5, Lines 3—8**
**Methods**			
**Study design**	4	Specify the study design in the methods section with a commonly used term (e.g., cross-sectional or longitudinal).	**Page 5, Line 20**
	5a	Describe the questionnaire (e.g., number of sections, number of questions, number and names of instruments used).	**Page 5, Lines 26—33**
**Data collection methods**	5b	Describe all questionnaire instruments that were used in the survey to measure particular concepts. Report target population, reported validity and reliability information, scoring/classification procedure, and reference links (if any).	**Page 5, Lines 33—43**
	5c	Provide information on pretesting of the questionnaire, if performed (in the article or in an online supplement). Report the method of pretesting, number of times questionnaire was pre-tested, number and demographics of participants used for pretesting, and the level of similarity of demographics between pre-testing participants and sample population.	**Page 5, Lines 39—43**
	5d	Questionnaire if possible, should be fully provided (in the article, or as appendices or as an online supplement).	**Page5, Line 43**
**Sample characteristics**	6a	Describe the study population (i.e., background, locations, eligibility criteria for participant inclusion in survey, exclusion criteria).	**Page 6, Lines 6 and 7**
	6b	Describe the sampling techniques used (e.g., single stage or multistage sampling, simple random sampling, stratified sampling, cluster sampling, convenience sampling). Specify the locations of sample participants whenever clustered sampling was applied.	**Page 6, Lines 7—13**
	6c	Provide information on sample size, along with details of sample size calculation.	**Page 6, Lines 14—21**

	6d	Describe how representative the sample is of the study population (or target population if possible), particularly for population-based surveys.	**Page 6, Lines 14—21**
**Survey administration**	7a	Provide information on modes of questionnaire administration, including the type and number of contacts, the location where the survey was conducted (e.g., outpatient room or by use of online tools, such as SurveyMonkey).	**Page 5, Line 21**<br><br>**Page 5, Line 44**
	7b	Provide information of survey's time frame, such as periods of recruitment, exposure, and follow-up days.	**Fully met**
	7c	Provide information on the entry process:<br>–>For non-web-based surveys, provide approaches to minimize human error in data entry.<br>–>For web-based surveys, provide approaches to prevent "multiple participation" of participants.	**Page 5, Line 21**<br><br>**Page 6, Line 13—14**
**Study preparation**	8	Describe any preparation process before conducting the survey (e.g., interviewers' training process, advertising the survey).	**Page 6, Line 9**
**Ethical considerations**	9a	Provide information on ethical approval for the survey if obtained, including informed consent, institutional review board [IRB] approval, Helsinki declaration, and good clinical practice [GCP] declaration (as appropriate).	**Page 5, Line 21—24**
	9b	Provide information about survey anonymity and confidentiality and describe what mechanisms were used to protect unauthorized access.	**Page 5, Lines 44—50**
**Statistical analysis**	10a	Describe statistical methods and analytical approach. Report the statistical software that was used for data analysis.	**Page 6, Lines 28—34**
	10b	Report any modification of variables used in the analysis, along with reference (if available).	**Page 6, Lines 30—33**
	10c	Report details about how missing data was handled. Include rate of missing items, missing data mechanism (i.e., missing completely at random [MCAR], missing at random [MAR] or missing not at random [MNAR]) and methods used to deal with missing data (e.g., multiple imputation).	**Page 6, Lines 35**
	10d	State how non-response error was addressed.	**N/A**
	10e	For longitudinal surveys, state how loss to follow-up was addressed.	**N/A**
	10f	Indicate whether any methods such as weighting of items or propensity scores have been used to adjust for non-representativeness of the sample.	**Page 6, line 38**
	10g	Describe any sensitivity analysis conducted.	**Page 6, line 37**
**Results**			
**Respondent characteristics**	11a	Report numbers of individuals at each stage of the study. Consider using a flow diagram, if possible.	**N/A**
	11b	Provide reasons for non-participation at each stage, if possible.	**N/A**

	11c	Report response rate, present the definition of response rate or the formula used to calculate response rate.	**Page 7, Lines 8—12**
	11d	Provide information to define how unique visitors are determined. Report number of unique visitors along with relevant proportions (e.g., view proportion, participation proportion, completion proportion).	**N/A**
**Descriptive results**	12	Provide characteristics of study participants, as well as information on potential confounders and assessed outcomes.	**Table 1**
**Main findings**	13a	Give unadjusted estimates and, if applicable, confounder-adjusted estimates along with 95% confidence intervals and p-values.	**Page 8, line 14, and line 19**
	13b	For multivariable analysis, provide information on the model building process, model fit statistics, and model assumptions (as appropriate).	**N/A**
	13c	Provide details about any sensitivity analysis performed. If there are considerable amount of missing data, report sensitivity analyses comparing the results of complete cases with that of the imputed dataset (if possible).	**N/A**
**Discussion**			
**Limitations**	14	Discuss the limitations of the study, considering sources of potential biases and imprecisions, such as non-representativeness of sample, study design, important uncontrolled confounders.	**Page 11, Lines 19—36**
**Interpretations**	15	Give a cautious overall interpretation of results, based on potential biases and imprecisions and suggest areas for future research.	**Pages 10 and 11, Lines 48—59 and Lines 3—12**
**Generalizability**	16	Discuss the external validity of the results.	**Page 9, Lines 21—30**
**Other sections**			
**Role of funding source**	17	State whether any funding organization has had any roles in the survey's design, implementation, and analysis.	**Page 11, Lines 57**
**Conflict of interest**	18	Declare any potential conflict of interest.	**Page 11, Lines 53—57**
**Acknowledgements**	19	Provide names of organizations/persons that are acknowledged along with their contribution to the research.	**Page 2, Lines 12, 17—23, and 31—33**

Supplementary S-3: Table 5, Reasons to assess for frailty

Table 5: Reasons to assess for frailty during PR

**Reasons to assess for frailty during PR**	**N=89**
**Availability of Normative data**	3 (3%)
**Easy equipment access**	5 (6%)
**Easy to administer**	11 (12%)
**Easy therapist understanding**	14 (16%)
**Easy patient understanding**	9 (10%)
**Evidence-based**	5 (6%)
**Freely licensed**	5 (6%)
**Low cost**	13 (15%)
**No training needed**	6 (7%)
**Protocol at centre**	8 (9%)
**Sensitive to PR change**	4 (4%)
**Technology integration**	2 (2%)
**Translations available**	0 (0%)
**Commonly used**	5 (6%)
**Other**	3 (3%)

Abbreviations: PR, Pulmonary Rehabilitation**.**

Supplementary S-4: Aspects of Pulmonary Rehabilitation require adaptation for Frailty

Q 30: What aspects of pulmonary rehabilitation care do you feel require adapting as a result of people with Chronic lung disease having frailty? (Clustered)

1. Exercise prescription	2. Program Options, and Structure	3. Facility resources	4. Education	5. Assessment	6. Diet/Nutrition	7. Time	8. Staff	9. Transportation and Access	10. Safety	11. Guidelines	Other
Some exercises require adaptation to suit clients needs, but no different to varying for other patient groups	There needs to be community and home based programmes as well	Falls risk - exercise room at centre does not cater for frail patients - inappropriate chairs, nil rails for balance	Home set up / education.	Initial assessment and	Dietetics involvement for frail people in pulmonary rehab	A longer assessment time and also the need to have the carer or family menber with the patient to have collateral information.	Staff to supervise	As our PR is currently just hospital based which can be a barrier for people to attend (transport, parking, fatigue in just getting to the programme for frail individuals)	Safety around exercise and	Good guidelines for fraility included into the Pulmonary Rehab Guidelines	Would be helpful to have clarification of the definition of "frailty" to answer this questions accurately.
Additional balance exercises	Provision of walking aids for walking component	Equipment, space	More education	Medical input	Dietetics input to support energy levels for exercise involvement.	Time to reflect on learning outcomes for patient and their support person.	Involving the MDT with all aspects of PRP so that issues can be managed effectively	Transport and access to programmes.	Safe options of alternative excercises	Specific guidelines regarding exercise and management would be extremely helpful.	Frailty is a broad term - the physical frailty +/- sarcopenia is probably where PR should target. With this in mind then
Modifications as necessary of the basic strength exercises	Supervision may need to be increased	After their initial assessment the choice of facility is considered (Hospital, not Community) but that has barriers of limited resources - infrastructure and personnel. I am limited in hospital gym space that I share with other clinicians and their in/outpatients, I run a small group (up to 5 participants) alone so need to consider the client mix and their ability/frailty and my ability to manage each client plus the potential of adverse events.	Tailoring education appropriately	Screening for frailty and adapting intervention as appropriate. ?	Nutritional interventions are important	Length of assessments and classes	Availability of clinical pharmacist. Availability of clinical psychologist.	Transport and ease of access to course	Setting and safety. Setting them up to be independent with exercise can be difficult and they often need one on one assistance.	Having a consistent model of care	I think this is a better approach rather than trying to include frailty assessments and management within what is usually a busy program where clients' Sp02, HR and interval training also need to be monitored and managed.
Exercises - increased time spent on things such as balance and	Class structure	Set up (to ensure safety when exercising)	Education sessions	A consistent tool that everyone uses would also be helpful.	Also involvement of dietitian to improve nutrition and support	More time in the assessment clinics	Staff to patient ratios	Access into the hospital is difficult unless the patient has family or transport.			More network support so that patient can be referred on after assessing frailty.
Exercise training is important	Prolonged period in program or alternate programs I.e lungs in action.	Aerobic and equipment use (treadmill and bike) are often inappropriate in my community setting for frail patients	Education needs to be modified if there is cognitive impairment present.	An approved frailty assessment.	There is minimal Dietetic in put currently and lots of Physio input, to manage frailty more comprehensively more Dietetic involvement is necessary.	Management - we are only allowed to have clients on service but these clients need sometimes longer than an 8 week program so this can be hard to manage	Patient to clinician ratio when increased numbers of patients with high level frailty	It might be worth noting also our programme can be difficult to get to because parking is a nightmare for patients (outpatient hospital with inadequate parking facilities) let alone frail patients experiencing SOBOE. We did also provide community classes for patients at one stage which seemed much easier to get to and we had good patient feedback about this also			Lack of referral options in the community for frail patients post Pulm rehab
Intensity and frequency of exercise needs to be modified	Determining alternative means of accessibility for example home based pulmonary rehabilitation as patients with chronic lung conditions and frailty tend to attend the hospital/community based program less frequently.	Improving accessibility to hospital/community based pulmonary rehabilitation (increase funding to staff additionally classes for ACT - currently only one provider in ACT) to ensure patients with frailty are reviewed in the class in a timely manner	Changing of education to meet needs of those that attend	Identification of these patients.	Nutrition	Time and increase therapist involvement for education, referrals	Needing more staffing.				Our programs are designed for those that are frail so despite not specifically measuring frailty our programs address frailty. Most of our outcome measures (from the toolkit) assess frailty either indirectly or directly
They take longer to complete the session. Closer supervision required for patients getting on to exercise bikes and walking on treadmills, moving around the gym with their O2 cylinders. More likely to experience physical exhaustion and therefore attendance at classes can be less likely. Reduced appetite often leading to less energy available to complete class.	Our PR programs are fixed 8 week courses. Frail clients need more flexibility as they are often unable to attend because of sickness, issues because of comorbidities and family issues.	We really don't have enough computers in our department to be able to provide a regular telehealth option/programme although this should definitely be a consideration going forwards for patients unable to attend a physical programme.	Education	Valid assessment tool for frailty (an appropriate one and not taking up too much assessment time)	Whether there would be a bigger push for having other health care professionals involved in their care and educating (e.g. dietician)	There may be a need for a slower stream of rehab where clients are involved for a longer period of time as they come from a lower level of function,	Class sizes and staff ratio to allow for closer supervision for those with frailty				Need to endure we select clients appropriately for PR vs management / slow stream palliation.
Targeted exercise programs for strength/endurance are already provided. Balance components could be integrated in to our exercise programs more.	Currently these clients are referred to a separate falls and balance class should the need be identified (however it is often not identified).	Exercise equipment and capability to use	Educational components.	Is there a way to do this as part of routine assessments/ even prior or to calculate it based on the information gathered in assessments.	Clients may need referral to other health professionals to assist them where they have greater needs eg dietitians.	More time both in terms of the session length and overall program length. Our program runs as closed groups. We have 5 intakes a year. Each program is 6 weeks in duration with participants attending 2 x 2 hour sessions a week. Due to the increased inability to attend, many participants may only receive a few sessions.					
Exercise prescription	Modification to program to allow success - ie not set bar too high and provide emotional support as anxiety plays hug e role in achieving outcomes.	Equipment use	Education delivered in a manner appropriate to any cognitive impairments (eg. providing and repeating short key messages, providing written/pictorial adjuncts to education, encouraging support person from client's life to be present at education)	Tools for identifying those whoe need further assessment.	Nutritional interventions is important						
Adapting exercises to suit individual ability	Supervision during exercise	Equipment	Education about the role of nutrition in frailty/sarcopenia often not a huge focus of assessments at my workplace	Assessment tools for pre and post program							
Incorporating balance exercises	Flexibility on programme delivery to suit patients needs eg time, venue length of sessions and length of programme.	Program is run over a large area (walking track in public place) and 150m from the gym.	Education content: more focused education content for people with frailty i.e. vitamin D supplementation, benefits of walking aids, falls prevention - ? referral to Stepping On/Falls Prevention program.	The traditional 6minWT for pre/post assessments often does not capture the 'Bigger Picture' of what is happening in frail patients with chronic disease							
Balance component.	Some of the exercises and would need more supervision	Needs to all be in one place for this group.	Falls prevention even though they don't typically feature in a traditional pulmonary rehab program.								
Developing a chair based program with balance activities included	Needing more supervision in terms of management and training when someone has significant level of frailty.	Equipment	More time spent on education around the exercises and modifying exercise programme for patient.								
Benefits of chair based activities	Multi disciplinary focus on behavior change and management. Follow up and check in with these patients more regularly re: adherence to exercise and management at home.										
Mainly the exercise component gets modified to cater for the patients requirements.	Clients may need pre-rehab to build up their exercise tolerance to be able to particpate in PRP.										
Exercises to be communicated in an easy to follow manner.	Frail clients may require closer supervision in terms of their mobility and balance so this may be hard to provide in a large group situation										
Exercises to be personally tailored to the individual's need and level.	I don't think the traditional PR program is always appropriate to manage a frail client, there are often (from PT perspective) lower level balance/mobility issues that need to be addressed - I have the luxury of being able to refer these clients to a Rehabilitation Medicine service										
Adequate strength dosing especially when treating in the home, in a group environment and over Telehealth	Encourage and give confidence. D/c planning and post group follow up.										
Tailored exercises.											
The requirement to walk for 20 minutes is difficult for the frail. A list of alternate aerobic activities specifically for the frail would be useful											
Exercise prescription, balance, strength training, falls prevention											
Exercises											

Supplementary S-5: Priorities for future work

Q 31: If you could list one priority area for future investment/research regarding frailty in people with chronic lung diseases undertaking pulmonary rehabilitation, what would it be? (Clustered)

1. Training and Exercises	2. Assessment	3. Guidelines	4. Resources, staff,Easy access and resources	5. Various Specialised Input	6. Program structure	Other
Workforce training so the people involved in the physical assessments/exercise training (in our programme the physiotherapists) have an understanding of frailty and know how to assess and adapt the standardised programme to meet these patients needs (patient centred care)	Best assessment / screening tool which is not too time-consuming to complete.	Guidelines for management.	Ease of access to an appropriate programme tailored to an individuals needs	Exercise involvement - recommendation of exercise from geriatricians and GP to assist frail people	One to one support for clients with frailty for a couple of sessions before the program starts.	Frailty and reversibility of this syndrome are important particularly with respect to presurgical considerations (e.g. transplantation candidacy) and or implications of frailty in terms of PR outcomes
Workforce training	Appropriate time efficient assessment tools i.e. questionnaires	Good guidelines for fraility in P Rehab	More resources allocated to assessment and management of frailty	Including more MDT input into PRP, for holistic, client centred care	Home based pulmonary rehabilitation programs	How to attract more Maori into the PR programme.
Workforce preparation/training	How would assessing for frailty change the way patients are managed in the Pulmonary Rehabilitation program?	Frailty treatment for patients with chronic lung disease	Program durations for those who are frail	Benefit of MDT and early collaborative intervention	Exercise and education is modified accordingly for patients to get maximum benefit. a real advanage of having individulised plans for each person attending.	It would be good to have information on patient outcomes in this group of patients.
Workforce training ie professional development	Development of a frailty assessment tool	If frailty is the main issue for the patient, potentially they would benefit from a frailty specific program instead of PR.	More time to educate on and implement changes	Nutrition and frailty	Understand that a generic PR program may not address the complexity of frailty.	Long term effects on frailty/falls/hospitalisations following pulmonary rehabilitation.
Workforce training to incorporate it more efficiently within our programs. We can always do better and I think this is important	There also seem to be so many outcome measures that can be utilised for assessing frailty and different papers seem to have assessed different outcomes measures. We rely a lot on research from other countries currently I find, especially in frailty in lung transplant (which is my main area of work)	Any differences in outcomes, or differences in preferences of models of care (centre based / home based etc)	Staffing	To manage frailty more comprehensively more Dietetic involvement is necessary.	Theoretically provision of generalized exercise in the PR setting will also address the mobility and exercise needs of these patients.	Medication reconciliation
More training and updates to be available to our team	Quick and easy idendifiers of frailty	We have a fantastic program who accepts various types of clients. It is common those on oxygen are frail due to limited confidence to exercise alone. We adapt our programs to suit individual limitations and slowly build on these. We assess for malnutrition, falls, pressure area, breathing, anxiety and depression....so another assessment would be a frustration. What can we do with the frailty assessment and what options are there to change our current practice if they score frail or not?	Staff patient ratio safety			
Staff training in identifying or screening for frailty	Although we do not use any frailty measures, during the nursing/physio assessment it is often clear of a persons frailty.	Flow charts of how to create a safe plan for the patients.	Access for patients to be referred onto appropriate services as required.I7:J7			
More training	Better assessment tolls and education re: management.	Clinical guidelines				
Workforce training	Creation of assessment tools and online training					
Training of individuals running Pulm Rehab.	Robust assessment and testing, physical and psychological, that could help determine and improve engagement levels so we target those who are most likely to benefit and be able to prioritise them into group vs Home Based PR vs normal care with Primary Care.					
Increased clinician awareness: Workforce preparation/training, seminars, online modules etc	Standardised assessment tool.					
Workforce training with regard to identification and assessment	The tools to screen for frailty					
Workforce training	Wider knowledge of the most appropriate assessment tools for frail patients with chronic respiratory disease					
Online training						
Workforce education on management						
Understanding Ax and how to adapt exercise and prescription based on assessment.						
Clinician education.						
Training program easily accessible for health professionals						
Workforce preparation and training including information in this area.						
I think workforce preparation/alignment to patient centred care with						

Supplementary S-6: Free comments or insights

Q 32: Please provide any free comments or insights regarding frailty in people with chronic lung disease and pulmonary rehabilitation in the text box below (optional). (Clustered)

1. Resourses, Transport and Time	2. Assessment	3. Insights	Other
Involvement of the MDT throughout the program from assessment to classes is required if we are to make any long term change.	Effective assessment of client needs to identify those most at risk is essential.	Our approach is to allocate clients with frailty to a more targeted program so that their strength, balance and stamina can be addressed and then they are referred onto PR2. The risk of falls can be quite high during the walking component of our PR2 classes especially as fatigue sets in.	Not all people with lung disease are frail.
Transport is a huge issue in our rural area, particular for frail people.	I routinely assess for frailty in a lung transplant candidate population and use a modified version of the Fried frailty phenotype for this. I assess 5 domains currently, physical exhaustion, appetite, grip strength, gait speed and physical activity levels. I would love to gain more knowledge as to what are the outcome measures recommended for use in PR. I also feel like components of the SPPB e.g balance assessments and components of the FFP such as gait speed (mobilise 5m in less than 6 seconds) are easily achieved in the lung transplant candidate population that I have so far assessed with end stage lung disease and I wonder if there are outcome measures for frailty that might be more applicable to a younger population partaking in pulmonary rehabilitation programmes who might then be going on to lung transplantation. Are certain outcome measures assessing frailty recommended more depending on the underlying lung disease or does this matter?	I refer patients to Pulm Rehab as a Resp Clinical Nurse Specialist and on discussion with some of the patients I have referred who are more frail they advise they didn't keep attending as found it too hard. I have then had to liaise with the physiotherapists running the exercise component (I only do 2 of the education sessions in the 8 week programme) to tailor and decrease the intensity of the exercise plan to meet the person's individualised level of function and need to try and re-engage them in pulm rehab which has helped. For those too frail to attend our outpatient centre at the hospital I refer to Community Pulm Rehab at home however this is only exercise and not a full education/exercise Programme run by the Community based Physiotherapists	There is the issue of overweight in this population so the 2 groups needs separate attention from a dietetic perspective.
Our program is already not funded with long waitlists. While addressing frailty specifically may be useful, it would be difficult from a time management perspective to add any additional assessment and management items without additional resourcing. Many frail clients decline pulmonary rehab referrals due to difficulties with endurance and clinic access, so are not accessing the service in the first place. Other models of care involving home programs/telehealth are potentially useful however once again funding is required to implement these at our site.	The number of assessment tools that clinicians need to give to patients in PR is exhausting i.e. CFS, SGRQ, PROMIS-29, CAT, HAD, FACIT. I feel that patients do not enjoy filling in multiple questionnaires that often repeat questions and clinicans likewise spend alot of time processing questionnaires instead of spending time providing education/motivational interviewing. The information is valuable but you need a workforce that is adaquately funded and staffed so that completing 6 questionnaires during a patient's initial and review assessment does not then impact on the amount of time a clinician spends with a patient asking the patient what they feel is the most important thing to them.	The social interaction of the clients within the program cannot be underestimated, many are lonely and isolated. Friendships have frequently developed in our groups and the ability to join a "post PRP ex group" has enable these clients to make even further gains	We do the best we can in our program but for someone who is very low level they often miss many sessions and can find the 2 hour sessions very long. (One hour education followed by one hour of individualized exercise.)
Currently although we have access to Physio support we don't have regular physio attendance at our group to to monitor and manage frailty. We can usually tell who are the ones who are most frail and at risk of falls but a management strategy would be useful		I have found that when people become too frail it becomes too hard for them to attend a community based programme. There are transport issues as well as fatigue. This is why an exercise programme although in theory should make a difference does not do as well for this group. They need a less intense programme over a longer duration to allow them enough time to build up their exercise capacity. More options eg start with home based use It to support if appropriate and build up to a more conventional community based programme so that they get the benefit of meeting others with similar problems	
Need more hours for better access to Physiotherapy input/dietician input		Enhancing engagement with the frail client is key - can be improved with an experienced Psychologist employing Motivational Interviewing prior to the program beginning.	
Travel to attend pulmonary rehab programs also a barrier for clients with frailty. And this is felt mostly in regional centres.		Home exercise programs (HEP) can be very beneficial for the frail. I find fear of attending groups due to frailty/breathlessness is high in clients. Also embarrassment at groups due to their poor exercise tolerance. Non attendance is high due to these factors. Training on HEP would be great.	
		It can be difficult managing frailty in a traditional PR program and in fact in my experience (generic) PR is not the best program to address frailty (unless of course the frailty is predominantly pulmonary related) if the goal is to keep someone safe and well at home and reduce ED presentations it would be better referring the client to a MDT Rehab service if available.	

The End of Supplementary Materials
